# The dipeptidyl peptidase 1 inhibition landscape in bronchiectasis and its future direction

**DOI:** 10.1183/16000617.0213-2026

**Published:** 2026-10-07

**Authors:** James D. Chalmers, Raksha Jain, Anne E. O'Donnell, Marcus A. Mall, Patrick A. Flume

**Affiliations:** 1 Radcliffe Department of Medicine, University of Oxford, Oxford, UK; 2 Department of Internal Medicine, University of Texas Southwestern Medical Center, Dallas, TX, USA; 3 Georgetown University Medical Center, Washington, DC, USA; 4 Department of Pediatric Respiratory Medicine, Immunology and Critical Care Medicine, Charité – Universitätsmedizin Berlin, Berlin, Germany; 5 German Center for Lung Research (DZL), associated partner site Berlin, Berlin, Germany; 6 German Center for Child and Adolescent Health (DZKJ), partner site Berlin, Berlin, Germany; 7 Cluster of Excellence ImmunoPreCept, Charité – Universitätsmedizin Berlin, Berlin, Germany; 8 Medical University of South Carolina, Charleston, SC, USA

## Abstract

Bronchiectasis (BE) is a heterogeneous lung disease characterised by chronic neutrophilic inflammation. A “vicious vortex” develops through the interactions between neutrophilic inflammation, infection and impaired mucociliary clearance, leading to progressive structural lung damage and worsening lung function. As part of this neutrophilic inflammation, neutrophil serine proteases (NSPs) overwhelm their natural inhibitors, with high NSP levels being associated with pulmonary exacerbations in people with BE. NSPs, activated by dipeptidyl peptidase 1 (DPP1) during neutrophil maturation in the bone marrow, are released from neutrophils at sites of inflammation. Pharmacological inhibition of DPP1 is a compelling approach to reduce neutrophilic inflammation in BE. Three DPP1 inhibitors have had favourable results in clinical studies for BE, namely brensocatib, florensocatib and verducatib, with recent approval of brensocatib, a first-in-class DPP1 inhibitor, in the US and Europe, providing a promising treatment option for people with BE. Many questions about DPP1 inhibition remain, including long-term efficacy and safety, and efficacy in people with cystic fibrosis, primary COPD and immunodeficiencies, who were generally excluded from prior large studies. Despite the availability of cystic fibrosis transmembrane conductance regulator modulator therapy, many people with cystic fibrosis continue to have pulmonary exacerbations. The inclusion of a broad population with a range of aetiologies is key in the development of DPP1 inhibitors to support access to treatment for all those who could benefit. This review explores the current DPP1 inhibition landscape, discussing the clinical studies to date for these three novel DPP1 inhibitors and the evolution of DPP1 studies in BE.

## Shareable abstract

**DPP1 inhibitors reduce exacerbations and may slow progression of bronchiectasis, validating inflammation as a key therapeutic target. Further investigation into their long-term efficacy, safety and treatment benefit across populations is needed.** https://bit.ly/3UnM9s1

## Introduction

Bronchiectasis (BE) is a chronic inflammatory lung disease and the third leading chronic lung disease worldwide [1, 2]. BE is radiologically confirmed by abnormally dilated bronchi and clinically characterised by cough, sputum production and lung infection [2–5]. Clinical presentation of BE is heterogeneous, given its association with various aetiologies, including post-infection, COPD, asthma and genetic conditions such as cystic fibrosis (CF) and primary ciliary dyskinesia [6, 7]. However, the cause of BE is unknown in ∼40% of cases [6]. There are multiple common treatable traits for BE of all aetiologies, including in cases with unknown cause [8, 9].

BE is characterised by neutrophilic inflammation, combined with infection, impaired mucociliary clearance and progressive structural lung damage, a “vicious vortex” that can lead to declining lung function, frequent pulmonary exacerbations (PExs) and disease progression [7, 10–12]. Neutrophils sustain this “vicious vortex” through a failure of normal phagocytic clearance of pathogens and the formation of pathogenic neutrophilic extracellular traps that release toxic granule products such as neutrophil serine proteases (NSPs) and azurocidin-1 into the airway [11, 13–19]. Imbalance between key NSPs (neutrophil elastase (NE), cathepsin G and proteinase 3) and their natural inhibitors leads to tissue destruction [11, 14, 20, 21].

PExs are a common manifestation of BE [3]. Significant increases in NSPs have been observed during BE PExs [22], with sputum NE being directly correlated with PEx frequency and severity [20, 23, 24]. High levels of NSPs also have a negative impact on pulmonary function, with elevated NE activity being associated with lung function decline in people with BE [20, 24]. People with BE who exacerbate frequently have poor quality of life (QoL), a greater risk of hospitalisation and increased mortality [25]. In people with BE, symptom burden can also be a predictor of disease deterioration. There is also a significant economic burden for people with BE, with hospitalisation being a major contributor to costs, particularly in people who exacerbate frequently [26, 27].

The reduction of active NSPs to alleviate inflammation in the lungs is a viable treatment target for people with BE [20, 28–30]. Several potential pathways could be targeted to lower active NSP levels, such as neutrophilic migration in order to reduce neutrophil numbers in the lungs [31]; however, various drugs targeting actors of this migration have not succeeded in people with BE, CF or COPD, and may increase risk of infection [20, 32–37]. Direct NE inhibition has also been explored, although these trials did not progress past Phase II for BE [20, 38, 39]. This may be due to inadequate dosing or because inhibiting only one NSP does not provide sufficient efficacy for treatment for BE [20].

NSPs are activated by dipeptidyl peptidase 1 (DPP1), also known as cathepsin C, in the bone marrow during neutrophil maturation [40–43]. Given the integral role of DPP1 in NSP activation, pharmacologic inhibition of DPP1 is a compelling target to reduce neutrophilic inflammation in BE (figure 1) [20, 28]. DPP1 inhibitor development is informed by the genetic condition Papillon–Lefèvre syndrome (PLS), which is caused by loss-of-function mutations in both alleles in the *DPP1* gene [44–46]; this leads to a reduction in active NSPs, but does not lead to immunodeficiency in most people, suggesting a viable immunomodulatory target [45, 46]. Safety topics of interest for DPP1 inhibitors are informed by the phenotype of PLS [12, 47], which is characterised by diffuse palmoplantar hyperkeratosis and severe periodontitis [44, 46]. Therefore, skin and periodontal monitoring is carried out during trials of DPP1 inhibitors [28, 47–49].

**FIGURE 1:**
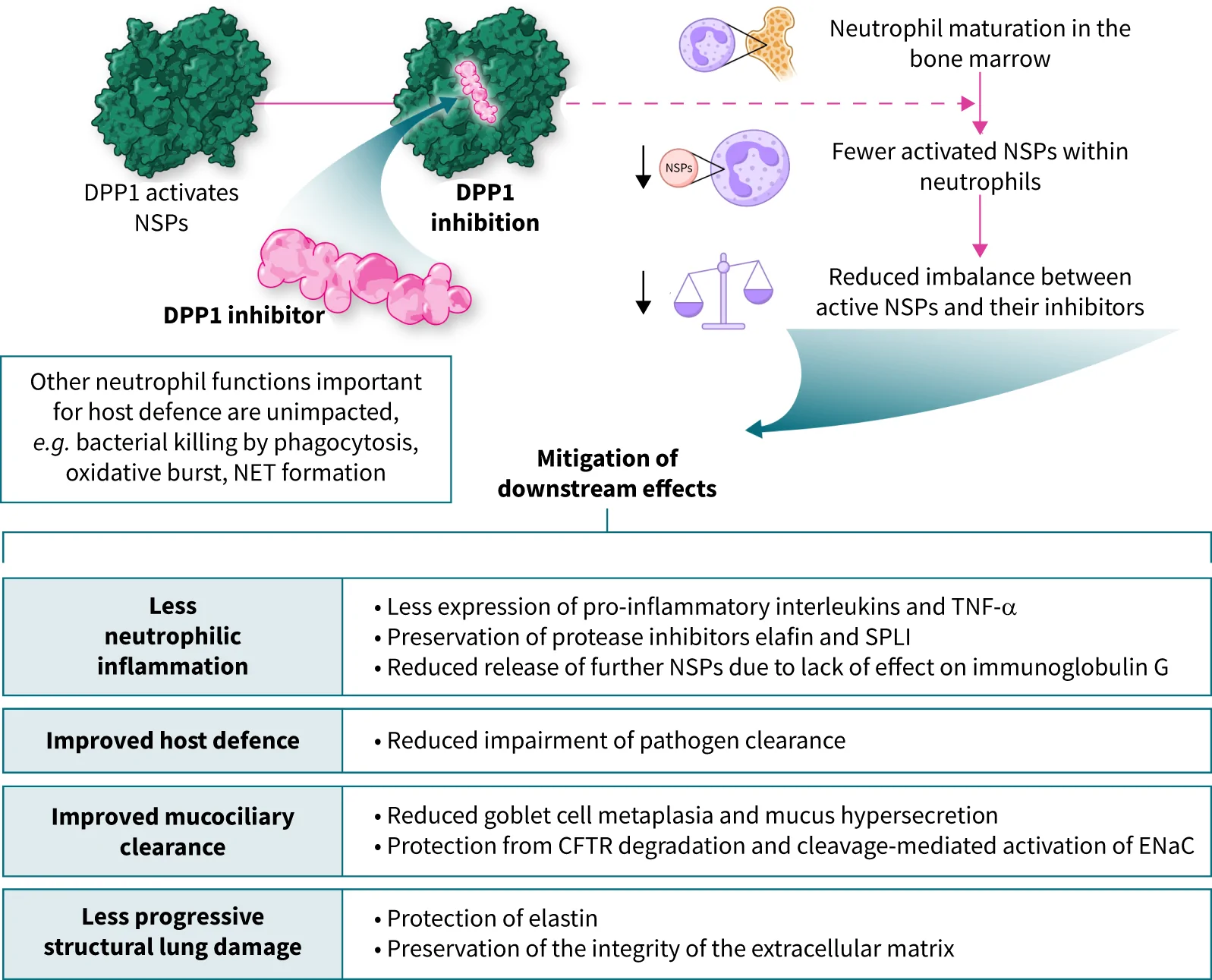
The mode of action of dipeptidyl peptidase 1 (DPP1) inhibition [11, 40–43, 71]. CFTR: cystic fibrosis transmembrane conductance regulator; ENaC: epithelium sodium channel; NET: neutrophil extracellular trap; NSP: neutrophil serine protease; SLPI: secretory leukocyte protease inhibitor; TNF-α: tumour necrosis factor-α.

Reducing NSP activity may reduce PEx frequency and severity, and improve lung function; it may also have a positive impact on mucus production and its properties [20, 23, 24, 50]. Increased NSP activity drives mucosal abnormalities, including NE-mediated pathological MUC5AC overexpression, mucus hypersecretion, impaired clearance and sustained airway inflammation [50, 51]. Currently, one reversible, potent and selective DPP1 inhibitor, namely brensocatib, is indicated for treatment of BE by regulatory authorities in the US and Europe, and two others, florensocatib and verducatib, are in clinical development, which could address this unmet need for people with BE (table 1) [28–30, 47–49, 52–57]. This review explores the current treatment landscape for DPP1 inhibition in BE, focusing on studies of these novel compounds.

**TABLE 1:** Summary of Phase II and Phase III clinical studies of dipeptidyl peptidase 1 (DPP1) inhibitors in people with bronchiectasis

Compound	ClinicalTrials.gov ID (study name where applicable)	Phase	Study completion	Study population	Randomised participants, n	Primary end-point
**Brensocatib**	NCT03218917 (WILLOW) [48, 95]	II	December 2019	NCFBE	256	Time to first PEx up to week 24
	NCT05090904 [61, 96]	IIa	November 2022	CF	29	Various PK end-points at days 1 and 28, and number of people with a TEAE up to week 8
	NCT04594369 (ASPEN) [47, 97]	III	October 2024	NCFBE	1721	Annualised rate of PExs up to week 52
**Florensocatib**	NCT05601778 (SAVE-BE) [49, 98]	II	June 2024	NCFBE	226	Annualised rate of PExs up to week 24
	NCT06660992 (HOPE-BE)^#^ [57, 62]	III	January 2027 (estimated)	NCFBE	700	Annualised PEx frequency up to week 52
**Verducatib**	NCT05238675 (AIRLEAF) [28, 99]	II	May 2024	NCFBE^¶^	322	Time to first PEx up to week 48
	NCT05865886 (CLAIRAFLY) [53, 67]	IIa	October 2024	CFBE	22	Occurrence of TEAEs up to week 16
	NCT05846230 (CLAIRLEAF)^#^ [52]	II	March 2027 (estimated)	CFBE/NCFBE	268	Occurrence of TEAEs over the course of the trial
	NCT06872892 (AIRTIVITY)^#^ [54, 63]	III	October 2028 (estimated)	CFBE/NCFBE^¶^	1755	Annualised rate of PExs up to week 76

^#^: Study is ongoing. ^¶^: Including COPD and asthma. CF: cystic fibrosis; CFBE: cystic fibrosis-related bronchiectasis; NCFBE: noncystic fibrosis-related bronchiectasis; PEx: pulmonary exacerbation; PK: pharmacokinetic; TEAE: treatment-emergent adverse event.

## Overview

### Brensocatib

A significant development in the treatment landscape for BE has been the recent US Food and Drug Administration (FDA) approval and European Commission approval, following positive opinion from the European Medicines Agency, of the first-in-class DPP1 inhibitor brensocatib for people with BE, which has demonstrated efficacy and safety across international, randomised Phase II and III studies [29, 30, 47, 48, 55, 56]. WILLOW (NCT03218917) was a Phase II study of brensocatib conducted in 256 people with non-CF-related BE (NCFBE) [48]. Adults (aged 18–85 years) with computed tomography (CT)-confirmed BE and ≥2 PExs in the 12 months before screening were randomised 1:1:1 to receive brensocatib at a dose of 25 mg or 10 mg, or placebo once daily for 24 weeks [48]. Those with a primary diagnosis of COPD or asthma were excluded [48]. Prolongation in time to first PEx (primary end-point) and reductions in PEx rate (secondary end-point; numerical reduction for 25 mg) and dose-dependent reductions in sputum NSP activity were observed with both the brensocatib 25 mg and 10 mg doses across the treatment period *versus* placebo [48, 58]. For time to first PEx, the adjusted hazard ratio (HR) for brensocatib compared with placebo was 0.58 in the 10 mg dose group and 0.62 in the 25 mg dose group [48]. The incidence-rate ratio for brensocatib compared with placebo was 0.64 in the 10 mg group and 0.75 in the 25 mg group [48].

Promising findings from WILLOW informed the design of the pivotal Phase III ASPEN study (NCT04594369) [47]. In total, 1721 participants with NCFBE, of whom 41 were adolescents (aged ≥12–<18 years, with a body weight of ≥30 kg at screening), were randomised 1:1:1 (2:2:1 for adolescents) to receive one of three treatments that were assessed in the WILLOW study (brensocatib 25 mg or 10 mg, or placebo) for 52 weeks [47]. Consistent with WILLOW, ASPEN participants were required to have CT-confirmed BE [47]. In the 12 months prior to screening, adults needed to have had ≥2 PExs and adolescents ≥1 PEx [47]. The annualised rate of PExs (primary end-point) and time to first PEx (secondary end-point) were reduced with both the brensocatib 10 mg and 25 mg doses *versus* placebo [47]. The annualised rate of PExs was 1.02 for the 10 mg dose group, 1.04 for the 25 mg dose group and 1.29 for the placebo group [47]. For time to first PEx, HR was 0.81 for the 10 mg dose group and 0.83 for the 25 mg dose group [47]. Additionally, the decline in forced expiratory volume in 1 s (FEV_1_) was less with brensocatib 25 mg than with placebo [47], the between-group difference in FEV_1_ at 52 weeks was 38 mL [47]. For adolescents, the HR for time to first PEx compared with placebo was 0.48 in the 10 mg dose group and 0.64 in the 25 mg dose group [59]. The annualised rate of PExs for adolescents was numerically reduced by 59% and 27% in the 10 and 25 mg dose groups, respectively, compared with placebo [59].

With regards to safety, in WILLOW, incidence of adverse events (AEs) (excluding PExs) was higher with either dose than placebo (63% in the 10 mg dose group, 54% in the 25 mg dose group and 38% in the placebo group) [48]. Hyperkeratosis was reported in one person who received placebo, in three people who received the 10 mg dose of brensocatib and in one person who received the 25 mg dose [48]. Incidence of periodontal disease was similar across all groups [48]. In ASPEN, for adults, higher rates of hyperkeratosis (3.0%) were reported in the 25 mg dose group, compared with the 10 mg (1.4%) and placebo (0.7%) groups [47]. In adolescents, AEs were similar across treatment groups and consistent with overall ASPEN results [59].

People with CF, primary diagnoses of COPD or asthma, and those on active nontuberculous mycobacterial treatment were excluded from ASPEN [47, 60]. However, in a separate study in people with CF who received 4 weeks of brensocatib treatment (10, 25 or 40 mg), pharmacokinetic profiles were comparable to those seen in people with NCFBE and reduced blood and sputum NSP activity [61]. All brensocatib doses were well tolerated, with no new identified safety signals in people with CF [61].

Of note in ASPEN, the clinical effect of NSP inhibition, reflected by separation of Kaplan–Meier exacerbation curves, was shown to become apparent ∼16 weeks after the first dose of brensocatib [47], despite much faster onset of inhibition of NSP activity (from approximately 3 weeks) in the airways [48]. Reasons for this are unclear; however, it is thought that this delay may be due to the time needed for the regeneration of airway epithelial cells, re-equilibration of the entire vicious vortex, improvement in the protease–inhibitor balance, effects of improved mucociliary clearance or the time delay to replace neutrophils with those impacted by brensocatib.

Findings from these two studies supported the approval of brensocatib by the FDA (August 2025) and in Europe (November 2025) for treatment of people with NCFBE aged ≥12 years [29, 30, 55, 56]. Brensocatib is the first and only current treatment for NCFBE that has been approved for use in the US and Europe [55, 56].

### Florensocatib

The DPP1 inhibitor florensocatib (previously HSK31858) is in clinical development [49, 57]. The Phase II SAVE-BE study (NCT05601778) investigated annualised frequency of PExs following florensocatib treatment in 226 adults with NCFBE in China [49]. Participants aged ≥18 years with CT-confirmed BE, compatible respiratory symptoms (cough, purulent or mucopurulent sputum or recurrent respiratory infections), ≥2 PExs in the past 12 months before screening, body mass index ≥18 kg·mg^−2^ and without primary diagnoses of COPD or asthma were randomised 1:1:1 to receive florensocatib 40 mg or 20 mg, or placebo once daily for 24 weeks [49]. Florensocatib decreased PEx frequency compared with placebo (primary end-point); mean annualised PEx frequency (per person-year) was 1.00 in the 20 mg dose group, 0.75 in the 40 mg dose group and 1.88 in the placebo group [49]. Regarding secondary efficacy end-points, time to first PEx was improved for both doses compared with placebo [49]. The HRs for the 20 mg dose and the 40 mg dose groups were 0.43 and 0.33, respectively [49]. There was a numerical improvement in least-squares mean difference in FEV_1_ percentage predicted compared with the placebo group for the 40 mg dose group (0.43) [49]. The safety profile of florensocatib was similar to placebo [49].

Following promising results from the Phase II study, the Phase III HOPE-BE study of florensocatib (NCT06660992) has been initiated to assess efficacy and safety of the 40 mg dose of florensocatib in a larger number of people with NCFBE in China [57]. Overall, 700 adults with ≥2 PExs in the past 12 months before screening were randomised to receive treatment for 52 weeks [57]. However, those with a primary diagnosis of COPD or asthma were excluded [57]. The primary end-point was annualised PEx frequency up to week 52, and secondary end-points included time to first PEx and relative changes from baseline in FEV_1_, 24-h sputum weight/sputum purulence score and Respiratory Symptoms Scale of the Quality of Life Questionnaire for Bronchiectasis [57, 62]. Findings from HOPE-BE may support the marketing application for florensocatib within mainland China [57].

### Verducatib

The DPP1 inhibitor verducatib (previously known as BI 1291583) had promising results in a Phase II, randomised, multinational study (AIRLEAF; NCT05238675) [28], supporting the current Phase III study (AIRTIVITY; NCT06872892) [54, 63]. AIRLEAF evaluated optimal dosing, efficacy and safety of verducatib in people with NCFBE [28]. Three doses of verducatib (5.0, 2.5 and 1.0 mg) were assessed in 322 adults with CT-confirmed BE and a history of PExs treated with antibiotics in the prior year (either ≥2 PExs or 1 PEx and a St. George's Respiratory Questionnaire (SGRQ) symptoms score >40 at screening [28]; this strategy aligns with European Respiratory Society (ERS) guidelines for identifying people with BE at risk of PExs [28, 64]). Participants were randomised 2:1:1:2 to receive placebo or once-daily treatment with verducatib 1.0, 2.5 or 5.0 mg for a period of at least 24 weeks and up to 48 weeks [28]. Verducatib demonstrated a similar safety profile to placebo [28]. Incidence of AEs was generally similar across groups (placebo: 92.7%; 1.0 mg: 84.9%; 2.5 mg: 86.8%; 5.0 mg: 87.9%), including potentially mode-of-action-related AEs such as skin AEs (placebo: 6.4%; 1.0 mg: 3.8%; 2.5 mg: 5.7%; 5 mg: 11.2%) and periodontal AEs (placebo: 4.6%; 1.0 mg: 7.5%; 2.5 mg: 1.9%; 5.0 mg: 2.8%) [28]. Hyperkeratosis was reported in 2, 0, 2 and 4 people in the placebo, 1.0, 2.5 and 5.0 mg dose groups, respectively [28]. The study demonstrated a significant benefit of verducatib *versus* placebo on time to first PEx that was dose-dependent on the primary end-point (time to first PEx up to week 48) [28]. The 5.0 mg and 2.5 mg doses numerically reduced PEx risk *versus* placebo, with HRs of 0.71 and 0.66, respectively [28]. Numerical reductions in rate of PExs up to week 48 (secondary end-point) were seen in the 5.0 mg and 2.5 mg dose groups (adjusted event rate ratios *versus* placebo: 0.89 and 0.68, respectively) [28]. Consistent with findings from the Phase III ASPEN study, the effect on PExs was also shown to occur ∼16 weeks after receipt of the DPP1 inhibitor in AIRLEAF [28, 47].

The 2.5 mg dose of verducatib had similar or improved outcomes *versus* the 5.0 mg dose [28]. The 2.5 mg dose reduced the risk of experiencing a severe PEx within 48 weeks compared with placebo, with an HR of 0.12, whereas no treatment benefit was seen for 5.0 mg [28]. With regards to patient-reported QoL, the 2.5 mg dose had the largest numerical effect on SGRQ symptoms score at week 24 (absolute change from baseline *versus* placebo adjusted mean for 2.5 mg: −3.33; 5.0 mg: −2.29; 1.0 mg: −1.58) and for SGRQ total score, the percentage of responders (decrease from baseline in SGRQ total score of ≥4 points) was greatest in the verducatib 2.5 mg group at both weeks 24 (2.5 mg: 62.7%; 5.0 mg: 51.5%; placebo: 47.6%; 1.0 mg: 45.1%) and 48 (2.5 mg: 75.8%; 1.0 mg: 55.9%; 5.0 mg: 55.6%; placebo: 47.7%) [28]. The 2.5 mg dose also led to the greatest change in FEV_1_ (adjusted mean *versus* placebo for 2.5 mg: 52.4 mL; 1.0 mg: 22.9 mL; 5.0 mg: 6.6 mL) at week 24 (with a nominally similar effect of 2.5 mg at week 48) and forced vital capacity (FVC) in mL from baseline to week 24 (adjusted mean *versus* placebo for 2.5 mg; 80.1 mL; 1.0 mg; 29.7 mL; 5.0 mg; 17.2 mL), with change in FVC being more pronounced at week 48 (2.5 mg: 132.0 mL) [28]. The 1.0 mg dose of verducatib was identified as subtherapeutic due to little evidence of benefit over placebo [28]. This evidence supports continued clinical testing of verducatib 2.5 mg. Verducatib can be administered at lower doses than brensocatib, likely due to its higher potency and more favourable distribution to the bone marrow (*in vivo* studies in mice) [65]. In an in-house analysis of exposure to brensocatib and verducatib, at ∼5 h after dosing at 0.1, 0.5 and 5.0 mg·kg^−1^, brensocatib showed lower bone marrow-to-plasma exposure ratios than verducatib (brensocatib: 28, 4 and 2, respectively; verducatib: 122, 81 and 32, respectively) [65]. This lower dosing, while maintaining higher bone-marrow-to-plasma exposure [65], is expected to minimise peripheral exposure and, therefore, potential systemic and peripheral AEs, while maintaining activity at the target site of action.

Results from AIRLEAF were consistent with findings from preclinical and Phase I studies of verducatib [65, 66]. Preclinically, verducatib was shown to dose-dependently inhibit NE and proteinase 3 in an *in vivo* lipopolysaccharide-challenge model in mice [65]. Across five Phase I trials in healthy volunteers, verducatib demonstrated a favourable safety and tolerability profile, with the majority of AEs being of mild or moderate intensity, and no serious AEs reported; verducatib also inhibited DPP1 in a dose-dependent manner [66].

Verducatib was tested in a Phase IIa trial in people with CF. The CLAIRAFLY study (NCT05865886) was conducted in 22 adults with CF-related BE (CFBE), who received 5 mg verducatib or placebo for 12 weeks [53, 67]. CLAIRAFLY will be discussed in the “CFBE and CF landscape” section below. CLAIRAFLY was developed as a response to the unmet need in this population, given that key clinical efficacy studies of DPP1 inhibition in BE have excluded people with CFBE [47–49, 57, 62]. People with CFBE have treatable traits seen in NCFBE and the verducatib development programme aims to address this need [8, 9, 68–71].

The ongoing Phase II CLAIRLEAF rollover study (NCT05846230) enrolled participants from AIRLEAF or CLAIRAFLY who completed treatment according to plan; participants continue the dose received in the parent trial and those who received placebo are randomised to receive 1.0, 2.5 or 5.0 mg for up to 52 weeks [52]. Initial results from a pooled analysis of AIRLEAF and CLAIRLEAF up to week 104 demonstrated consistently positive effects of the verducatib 2.5 mg dose and a safety profile consistent with AIRLEAF, supporting the long-term efficacy and safety of verducatib in people with NCFBE [72].

Given the promising results from Phase II studies of verducatib, the randomised Phase III AIRTIVITY study investigating the efficacy, safety and tolerability of verducatib 2.5 mg is being conducted in a large and broad population of people with BE, irrespective of underlying aetiology [63]. AIRTIVITY is the first Phase III DPP1 inhibitor study in BE to include people with CF [47, 57, 63]. It is expected that ∼1755 people with BE (including adults and adolescents) will be randomised 2:1 to receive verducatib 2.5 mg or placebo once daily for a treatment period of 52–76 weeks [63]. Consistent with AIRLEAF and in contrast to ASPEN, people with primary COPD and asthma are eligible for enrolment [28, 47, 63]; other populations generally excluded from prior DPP1 inhibitor trials are also eligible, including people with stable immunodeficiency conditions, and the trial is less restrictive in terms of prior medical treatments [47–49, 54, 57, 63]. Participants need to have CT-confirmed BE and a history of PExs requiring treatment with antibiotics in the year prior to screening (either ≥2 PExs in the past year, 1 PEx in the past year with an SGRQ score >40 at screening (adults only), or 1 PEx and high symptom burden according to the investigator's judgment (adolescents only)) [63]. The AIRTIVITY study's inclusion criteria are therefore aligned with the recently published ERS guidelines on adult BE, which consider people with ≥2 PExs in the prior year, 1 or more severe PEx, or 1 PEx plus severe daily symptoms to be at high risk [64]. The primary end-point of AIRTIVITY is annualised rate of PExs up to week 76, with secondary end-points including change from baseline in lung function and Quality of Life Questionnaire – Bronchiectasis respiratory symptoms domain score, time to first PEx and annualised rate of severe PExs *versus* placebo [63].

If the positive results seen for verducatib so far in Phase II trials are also observed in AIRTIVITY, these results should provide confirmatory evidence for using verducatib 2.5 mg in a broad population of people with BE, irrespective of aetiology. Additionally, participants who complete treatment as planned may have the option to continue into an open-label extension study.

### CFBE and CF landscape

CF is caused by dysfunctional cystic fibrosis transmembrane conductance regulatory (CFTR) protein and is characterised by abnormally viscous mucus, impaired mucociliary clearance and mucus plugging, with a cycle of chronic infection and neutrophilic inflammation leading to progressive structural lung damage [73, 74]. Most people with CF have BE, with children with CF developing BE early in life [73, 75]. In children with CF, the development of BE is associated with free NE activity in bronchoalveolar lavage fluid [75]. CFTR modulator therapy has become the gold-standard treatment for CF and the triple combination (elexacaftor/tezacaftor/ivacaftor; ETI) led to unprecedented improvements in the lives of people with CF with CFTR-responsive variants, improving lung function, respiratory symptoms, sweat chloride levels and PEx rates [73, 76–83]. However, approximately 5–11% of people with CF are ineligible for or intolerant of CFTR modulator therapy [84, 85]. According to the EU label, those with two Class I mutations, who are known to not express CFTR protein, are excluded from treatment with ETI [86]. Moreover, people with CF who are receiving CFTR modulator therapy have been shown to have persisting levels of airway inflammation and elevated levels of NE similar to people with NCFBE and may continue to suffer from PExs [69–71, 87–92]. Considering the above, people with CF may benefit from treatment targeting the neutrophilic inflammation element of their BE, including people treated with CFTR modulator therapy [69–71, 87, 88, 90]. Therefore, DPP1 inhibitors could help address this unmet need. However, the label for brensocatib does not include people with CF [29, 30].

In CLAIRAFLY, 22 adults with CFBE were randomised [53, 67]; other than restricting the study to include people with CF *versus* other aetiologies, CLAIRAFLY had similar inclusion criteria to AIRLEAF [28, 53, 67]. A comparison between people with NCFBE from AIRLEAF and people with CFBE from CLAIRAFLY demonstrated comparable verducatib exposure and overall safety profiles with verducatib 5 mg [67]. This suggests that the pharmacokinetics of DPP1 inhibition in people with CF are comparable to those with other aetiologies of BE. Together, these data support the continued investigation of verducatib in people with CF, for the first time in a Phase III study, as well as in people with NCFBE [67].

### Evolution of DPP1 clinical studies

The success seen with brensocatib, florensocatib and verducatib validates the concept and mode of action of DPP1 inhibition in BE treatment. Despite important results from the ASPEN trial, key questions remain. No statistically significant effect on severe PExs was observed in ASPEN [47] and prevention of these events is highly relevant. The impact of DPP1 inhibitors on symptoms is unclear, as the clinical relevance of changes seen in the ASPEN trial in symptom questionnaires was debated by the FDA. This is an important consideration for people with BE who prioritise improvement in QoL as a key treatment goal. He *et al*. [93] recently described currently unresolved questions around DPP1 inhibition in BE following the approval of brensocatib, including whether lung function can be conserved through early intervention, what are the long-term clinical benefits and safety beyond 1 year, and the substantial effects seen with lower doses and little additional benefit seen in higher doses. Additionally, the delay between bone marrow effects of DPP1 inhibitors and clinical benefit seen in clinical studies could suggest that it may be beneficial to implement early treatment after a PEx, rather than waiting for further events [93]. The longest treatment period (excluding expanded access studies) in any randomised controlled DPP1 inhibitor study to date is 52 weeks [28, 47–49, 57]. Given the late separation of the Kaplan–Meier curves for PEx and the important lung function benefit in ASPEN [47], it is important to establish whether sustained or increasing efficacy may be seen beyond 52 weeks.

The general impression of safety with DPP1 inhibitors is positive, with relatively few participant discontinuations across all studies [28, 47–49]. In ASPEN, 3% of people in the 25 mg dose group experienced hyperkeratosis [47], a side effect that, although unclear, may be related to the peripheral distribution of DPP1 inhibitors [94]. An important differentiator of verducatib is a high bone marrow-to-plasma concentration ratio [65], which is predicted, but not yet proven, to reduce the risk of hyperkeratosis and will need to be investigated in AIRTIVITY. AIRTIVITY includes participants with 1 PEx with a high symptom burden (SGRQ symptoms score >40 for adults or according to the investigator for adolescents), broadening the treatment to more people with BE, including people with CF, COPD and asthma. AIRTIVITY also provides a longer treatment period (at least 52 weeks and up to 76 weeks), in addition to the option for an open-label extension study [63]. Verducatib 2.5 mg has also been shown to reduce the risk of experiencing severe PExs in AIRLEAF [28]. Whether this is a differentiating effect of verducatib requires testing in AIRTIVITY. In addressing a broader population for a longer period, AIRTIVITY aims to substantially extend our understanding of the role of DPP1 inhibition as a long-term treatment for BE [63].

## Conclusion

There remains an unmet need in BE treatment. The approval of brensocatib by the FDA and in Europe changed the BE treatment landscape, providing an effective option for people with NCFBE to treat a key driver of their disease [29, 30, 55, 56]. Additionally, the DPP1 inhibitors verducatib and florensocatib are in clinical development, with the results of ongoing Phase III studies eagerly awaited [57, 63]. Confirmatory evidence for the efficacy and safety of verducatib in the large Phase III setting could support the use of DPP1 inhibition in a broader population of people with BE [63].

The success observed so far with DPP1 inhibitors represents a collective strength, validating the mode of action of DPP1 inhibition in BE and the therapeutic potential of this class of drug. Researchers should take confidence from these findings, as should people with BE, their families and caregivers, and their doctors, given the promising treatment that is now available. Further investigation into DPP1 inhibitors is warranted to validate the long-term efficacy and safety of these compounds, confirm the treatment benefit across subgroups that reflect the real-world population, and investigate if concomitant use with other treatments is feasible and effective.

## Expert opinion: Prof. James D. Chalmers

DPP1 inhibition represents a paradigm shift in the management of patients with BE. Treatments to date have predominantly focused on palliating symptoms (airway clearance, mucoactive drugs) or targeting infection with antibiotics. Even macrolides, the most effective current treatment, is an antibiotic class and runs the risk of driving antimicrobial resistance and has a poorly defined mechanism of action. As we think about an optimal treatment for BE, we would wish to have a treatment which can reduce the burden of exacerbations and symptoms, which addresses the inflammatory process that is central to the disease, and which can interrupt the “vicious vortex” of disease without carrying the long-term risks associated with repeated broad-spectrum antibiotic exposure. DPP1 inhibition achieves these objectives. DPP1 inhibition therefore has the potential to optimise the management of patients with BE at high risk of exacerbations. The evidence from Phase II and Phase III trials on exacerbations is compelling, with approximate efficacy of 20–60% reduction on key exacerbation end-points across the different compounds. That all trials to date have met their primary end-points strongly validates the mechanism.

There are several compelling findings with DPP1 inhibitors worthy of discussion. First, DPP1 inhibition has a remarkable effect on the “vicious vortex” of BE. Translational research has shown a strong effect of DPP1 inhibition on reducing MUC5AC levels in sputum. Reductions in this key inflammatory mucin may underlie the clinical efficacy of DPP1 inhibition in BE, since mucus symptoms are dominant in the disease and are central to lung function decline. This effect on mucociliary clearance is supported by recently reported data from the CT sub-study of the ASPEN trial suggesting reduced mucus plugging. DPP1 inhibition enhances antimicrobial defence by upregulating the secretory leukocyte protease inhibitor and neutrophil defensins, as well as resetting the cytokine response. These effects likely underlie the beneficial effects on lung function decline seen with the 25 mg dose of brensocatib, and it was observed that 25 mg had a greater impact on MUC5AC than 10 mg.

The ability to prevent progression of disease, as measured by FEV_1_, FVC and in an exploratory analysis, and to reduce mucus plugging on CT, raises the potential of DPP1 inhibition as a disease-modifying therapy. Disease modification is defined as delaying, slowing or preventing the progression of a disease by targeting its underlying cause rather than merely alleviating symptoms. Progression of BE is not well defined, but most definitions would include changes over time in lung function or radiological appearance on CT, both of which are altered by DPP1 inhibitors. Longer-term studies beyond 1 year are likely needed to determine whether the apparent slowing of disease progression with these therapies is sustained.

Another remarkable observation is the consistency of effect across patient subgroups. In WILLOW, ASPEN, AIRLEAF and SAVE-BE, no “nonresponding subgroup” has been consistently identified and perhaps only patients from Asian countries have been proposed as super-responders. This appears to confirm the central role of neutrophilic inflammation in BE. The target is present in all patients and therefore the benefit is possible in all patient groups.

The potential disease-modifying nature of the treatment is, in my experience, important for patients who want a treatment that will make them feel better, prevent exacerbations, but also want a treatment that will prevent the disease from getting worse.

Nevertheless, this raises a number of difficult questions to answer. Where should DPP1 inhibition be positioned in relation to other therapies such as macrolides? What does success look like and at what stage should treatment success be evaluated? Upcoming guidelines that include DPP1 inhibition may address some of these questions, which are likely to be different in different healthcare systems. An important shift in thinking is needed towards an idea of disease activity, where the goal of treatment is to achieve disease control/stability, represented by a low risk of exacerbation, stable lung function and manageable symptoms.

My personal view is that for patients at high risk of exacerbations and progressive disease, DPP1 inhibition represents the most effective evidence-based therapy and has the potential to become the backbone therapy for “highly active disease”. The DPP1 inhibition landscape is evolving, as is our understanding of the disease. Next-generation DPP1 inhibitors will broaden our understanding of the role of these drugs by broadening the patient population in which they have been tested, the length of time over which they are tested and the end-points tested. This may allow more patients to have access to these therapies as well as strengthening the evidence of whether DPP1 inhibitors are genuinely disease-modifying. Any differentiation between the drugs is speculative and will need to be established in Phase III or real-life studies.

Taken together, DPP1 inhibition represents an exciting development for BE patients who continue to have a massive unmet need. These are new therapies and advancing the science in this area is critical if this new tool is to be used optimally.

**Questions for future research**

Further investigation into DPP1 inhibitors is warranted to:

- Validate their long-term efficacy and safety
- Confirm the treatment benefit and safety across subgroups that reflect the real-world population
- Investigate concomitant use with other treatments
- Determine the extent to which DPP1 inhibitors are disease modifying
- Evaluate DPP1 efficacy in subpopulations of BE, including CF, where data are currently lacking

## Supplementary material

**Please note:** supplementary material is not edited by the Editorial Office, and is uploaded as it has been supplied by the author. Supplementary material: interactive infographic ERR-0213-2026.SUPPLEMENT
