# Supplementary material for: The dipeptidyl peptidase 1 inhibition landscape in bronchiectasis and its future direction
Source: Eur Respir Rev. 2026 Oct 7;35(182):260213. doi: 10.1183/16000617.0213-2026 (PMC13643495; doi:10.1183/16000617.0213-2026)
Supplement: Supplementary file 1 [file ERR-0213-2026.SUPPLEMENT.pdf]

# The dipeptidyl peptidase 1 inhibition landscape in bronchiectasis and its future direction

Chalmers JD, Jain R, O'Donnell AE, *et al.* The dipeptidyl peptidase 1 inhibition landscape in bronchiectasis and its future direction. *Eur Respir Rev* 2026; 35: 260213. doi: 10.1183/16000617.0213-2026.

## Introduction to bronchiectasis and dipeptidyl peptidase 1 inhibition

**BE and DPP1 inhibition**

BE is a chronic lung disease characterised by **neutrophilic inflammation**[1–4]

Pharmacological **inhibition of DPP1** aims to suppress NSP activity[5–9]

To reduce PEx frequency and severity, and improve lung function in people with BE[7,10]

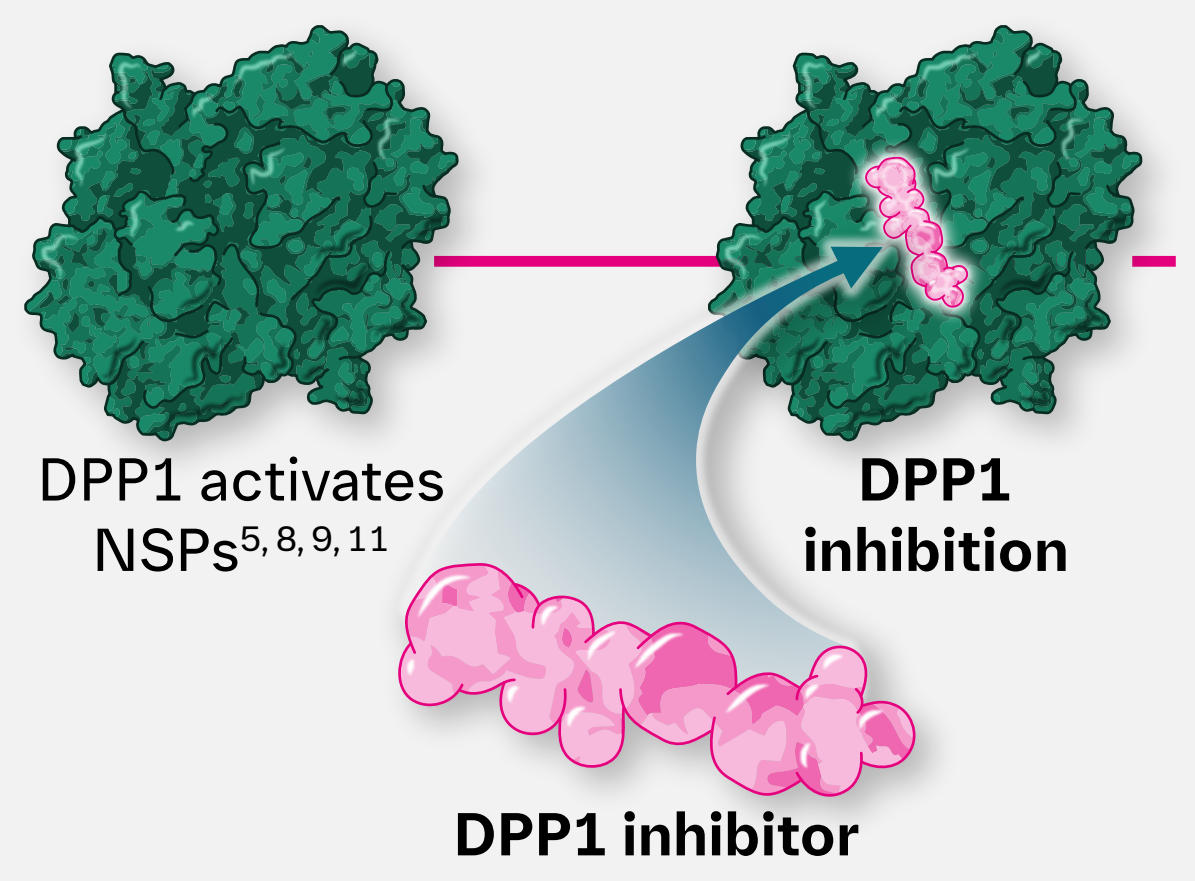


Neutrophil maturation in the bone marrow[10,11]
↓
Fewer activated NSPs within neutrophils
↓
Reduced imbalance between active NSPs and their inhibitors[2]

**Mitigation of downstream effects**

*Other neutrophil functions important for host defence are unimpacted*[5]

- ↓ Neutrophilic inflammation[12]
- ↑ Host defence[12]
- ↑ Mucociliary clearance[12]
- ↓ Progressive structural lung damage[12]

## DPP1 inhibitors in clinical development

| Compound | Phase | Study name | Study population | Doses | No. of pts. randomised | Treatment length (weeks) | Primary endpoint results |
|---|---|---|---|---|---|---|---|
| Brensocatib[17–19] (FDA & European Commission approved[13–16]) | II | WILLOW | NCFBE | 10 mg<br>25 mg | 256 | 24 | Increased time to first PEx for both doses |
| | IIa | NCT05090904 | CF | 10 mg<br>25 mg<br>40 mg | 29 | 4 | PK profiles in CF were comparable to those with NCFBE |
| | III | ASPEN | NCFBE | 10 mg<br>25 mg | 1,721 | 52 | Reduction in rate of PExs with both doses in adults |
| Florensocatib[20–22] | II | SAVE-BE | NCFBE | 20 mg<br>40 mg | 226 | 24 | Decreased frequency of PExs with both doses |
| | III | HOPE-BE* | NCFBE | 40 mg | 700 | 52 | Yet to be published (Estimated study completion: January 2027) |
| Verducatib[6,23–27] | II | AIRLEAF® | NCFBE† | 1 mg<br>2.5 mg<br>5 mg | 322 | 24–48 | Dose-dependent increase in time to first PEx |
| | IIa | CLAIRAFLY® | CFBE | 5 mg | 22 | 12 | Comparable safety between people with NCFBE (AIRLEAF®) and with CFBE (CLAIRAFLY®) |
| | II | CLAIRLEAF®* | AIRLEAF® & CLAIRAFLY® | 1 mg<br>2.5 mg<br>5 mg | 268 | 52 | Yet to be published (Estimated study completion: March 2027) |
| | III | AIRTIVITY®* | CFBE + NCFBE† | 2.5 mg | 1,755† | 52–76 | Yet to be published (Estimated study completion: October 2027) |

Click for **additional efficacy** and **safety** results

### CFBE and CF landscape

~5–11% of people with CF are **ineligible for or intolerant of CFTR modulator therapy**[28,29] + Those receiving CFTR modulator therapy have **persistent airway inflammation**[12,30–33] = People with CF may benefit from treatment **targeting the neutrophilic inflammation** element of their BE

**AIRTIVITY® is the first Phase III DPP1 inhibitor study in BE to include people with CF**[18,22,27]

## Evolution of DPP1 clinical studies

| Brensocatib & florensocatib studies[17,18,22,34] | Verducatib Phase III AIRTIVITY® study[6,27] |
|---|---|
| Exclusion of high-risk patients with low PEx frequency‡ | Includes people with **1 PEx** (that led to antibiotic treatment in the last 12 months before screening) and a high symptom burden§ at screening, as well as people with ≥2 PEx |
| **Lack subgroups** such as CF, primary COPD and asthma | Includes people with **CF, COPD and asthma** |
| **No significant effect on severe PEx** in the Phase III ASPEN study | Continuing to test the reduction in the **risk of experiencing severe PExs** seen in the Phase II AIRLEAF® study |
| Longest treatment period in any randomised controlled DPP1 inhibitor study (excluding expanded access studies) is **52 weeks** | Treatment period of **52–76 weeks,** with potential open-label extension study |

## Conclusion

The success of brensocatib, florensocatib, and verducatib studies **validates the DPP1 inhibitor approach in BE**

Click for expert opinion piece from **Prof. James D. Chalmers**

**Does DPP1 inhibition have the potential to optimise management of BE?**
**What insights do we have on DPP1 inhibition in BE?**
**What questions remain?**

*Study is ongoing
†Including COPD and asthma
§SGRQ Symptoms score >40 for adults or according to the investigator for adolescents
‡Eligible adolescents in the brensocatib Phase III ASPEN study had at least one exacerbation that led to antibiotic treatment in the 12 months before screening[18]

**Abbreviations**

AE, adverse event; BE, bronchiectasis; CF, cystic fibrosis; CFBE, cystic fibrosis-related bronchiectasis; CFTR, cystic fibrosis transmembrane conductance regulator; COPD, chronic obstructive pulmonary disease; CT, computed tomography; DPP1, dipeptidyl peptidase 1; FDA, US Food and Drug Administration; $FEV_1$, forced expiratory volume in 1 second; FVC, forced vital capacity; HR, hazard ratio; MoA, mode of action; NCFBE, non–cystic fibrosis-related bronchiectasis; NE, neutrophil elastase; NSP, neutrophil serine protease; PEx, pulmonary exacerbation; pts, participants; SGRQ, St. George's Respiratory Questionnaire.

**References**

**1.** Long MB, et al. Lancet Respir Med. 2024; 12(11):901–14; **2.** Oriano M, et al. Int J Mol Sci. 2021; 22(11):5996; **3.** Polverino E, et al. Eur Respir J. 2017; 50(3):1700629; **4.** Flume PA, et al. Lancet. 2018; 392(10150):880–90; **5.** Chalmers JD, et al. Front Immunol. 2023; 14:1239151; **6.** Chalmers JD, et al. Eur Respir J. 2025; 65(1):2401551; **7.** Chalmers JD, et al. Eur Respir J. 2025; 65(1):2401050; **8.** Chalmers JD, et al. Am J Respir Crit Care Med. 2017; 195(10):1384–93; **9.** Adkison AM, et al. J Clin Invest. 2002; 109(3):363–71; **10.** Gardiner P, et al. Br J Pharmacol. 2016; 173(15):2390–401; **11.** Keir HR, et al. Eur Respir Rev. 2022; 31(163):210241; **12.** Mall MA, et al. Eur Respir Rev. 2024; 33(173):240001; **13.** U.S. Food & Drug Administration. BRINSUPRI™ (brensocatib) prescribing information. https://www.accessdata.fda.gov/drugsatfda_docs/label/2025/217673s000lbl.pdf. Date last accessed: 5 November 2025; **14.** European Medicines Agency. Brinsupri : EPAR - Product information. https://www.ema.europa.eu/en/documents/product-information/brinsupri-epar-product-information_en.pdf. Date last accessed: 23 January 2026; **15.** Insmed. FDA approves BRINSUPRI™ (brensocatib) as the first and only treatment for non-cystic fibrosis bronchiectasis, a serious, chronic lung disease. https://investor.insmed.com/2025-08-12-FDA-Approves-BRINSUPRI-TM-brensocatib-as-the-First-and-Only-Treatment-for-Non-Cystic-Fibrosis-Bronchiectasis,-a-Serious,-Chronic-Lung-Disease. Date last accessed: 5 November 2025; **16.** Insmed. European Commission approves BRINSUPRI™ (brensocatib) as the first and only treatment approved for non-cystic fibrosis bronchiectasis in the European Union. https://investor.insmed.com/2025-11-18-European-Commission-Approves-BRINSUPRI TM-brensocatib-as-the-first-and only treatment approved for non-cystic fibrosis bronchiectasis in the European Union. Date last accessed: 23 January 2026; **17.** Chalmers JD, et al. N Engl J Med. 2020; 383(22):2127–37; **18.** Chalmers JD, et al. N Engl J Med. 2025; 392(16):1569–81; **19.** Konstan MW, et al. Clin. Pharmacokinet. 2025 64(10):1561–74; **20.** Zhong NS, et al. Lancet Respir Med. 2025; 13(5):414–24; **21.** ClinicalTrials.gov. A study to assess the efficacy and safety of HSK31858 in participants with non-cystic fibrosis bronchiectasis. https://clinicaltrials.gov/study/NCT06660992. Date last accessed: 23 January 2026; **22.** Zhong NS, et al. ERJ Open Res. 2026; in press: https://doi.org/10.1183/23120541.00182-2026; **23.** Mall MA, et al. J Cyst Fibros. 2025; 24:S35; **24.** ClinicalTrials.gov. A study to test how well BI 1291583 is tolerated by people with cystic fibrosis bronchiectasis (Clairafly™). https://www.clinicaltrials.gov/study/NCT05865886. Date last accessed: 26 January 2026; **25.** ClinicalTrials.gov. Clairleaf™: A study to test long-term treatment with BI 1291583 in people with bronchiectasis who took part in a previous study with this medicine. https://clinicaltrials.gov/study/NCT05846230. Date last accessed: 23 January 2026; **26.** ClinicalTrials.gov. The AIRTIVITY™ study: A study to find out whether BI 1291583 helps people with bronchiectasis. https://www.clinicaltrials.gov/study/NCT06872892. Date last accessed: 26 January 2026; **27.** Chalmers JD, et al. ERJ Open Res. 2026; in press: https://doi.org/10.1183/23120541.01068-2025; **28.** Burgel P-R, Mall MA. Eur Respir J 2026; 67: 2501783; **29.** Cystic Fibrosis Foundation. 2024 Patient Registry Annual Data Report. Bethesda, Maryland: Cystic Fibrosis Foundation; 2025; **30.** Schaupp L, et al. Eur Respir J. 2023; 62(2):2202153; **31.** Casey M, et al. Thorax. 2023; 78(8):835–9; **32.** Nussstein H, et al. Eur Respir J. 2025; 2026; 67(2):2500616; **33.** Sagel SD, et al. Ann Am Thorac Soc. 2025; online ahead of print: https://doi.org/10.1513/AnnalsATS.202507-817OC; **34.** Chalmers JD, et al. ERJ Open Res. 2024; 10(4):00151-2024; **35.** Maitre B, et al. Am J Respir Crit Care Med. 2025; 211:A7421; **36.** Cipolla D, et al. Respir Res. 2023; 24(1):133.

**Acknowledgements**

This interactive PDF was funded by Boehringer Ingelheim Pharmaceutical Inc. The authors meet criteria for authorship as recommended by the International Committee of Medical Journal Editors (ICMJE). The authors did not receive payment related to the development of the interactive PDF. Amy Bohm, MBio, of Nucleus Global, provided writing, editorial support and formatting assistance, which was contracted and funded by Boehringer Ingelheim. Boehringer Ingelheim was given the opportunity to review the interactive PDF for medical and scientific accuracy as well as intellectual property considerations.

# The dipeptidyl peptidase 1 inhibition landscape in bronchiectasis and its future direction

Chalmers JD, Jain R, O'Donnell AE, *et al*. The dipeptidyl peptidase 1 inhibition landscape in bronchiectasis and its future direction. *Eur Respir Rev* 2026; 35: 260213. doi: 10.1183/16000617.0213-2026.

## Introduction to bronchiectasis and dipeptidyl peptidase 1 inhibition

**BE and DPP1 inhibition**

BE is a chronic lung disease characterised by **neutrophilic inflammation**[1–4]

Pharmacological **inhibition of DPP1** aims to suppress NSP activity[5–9]

To reduce PEx frequency and severity, and improve lung function in people with BE[7,10]

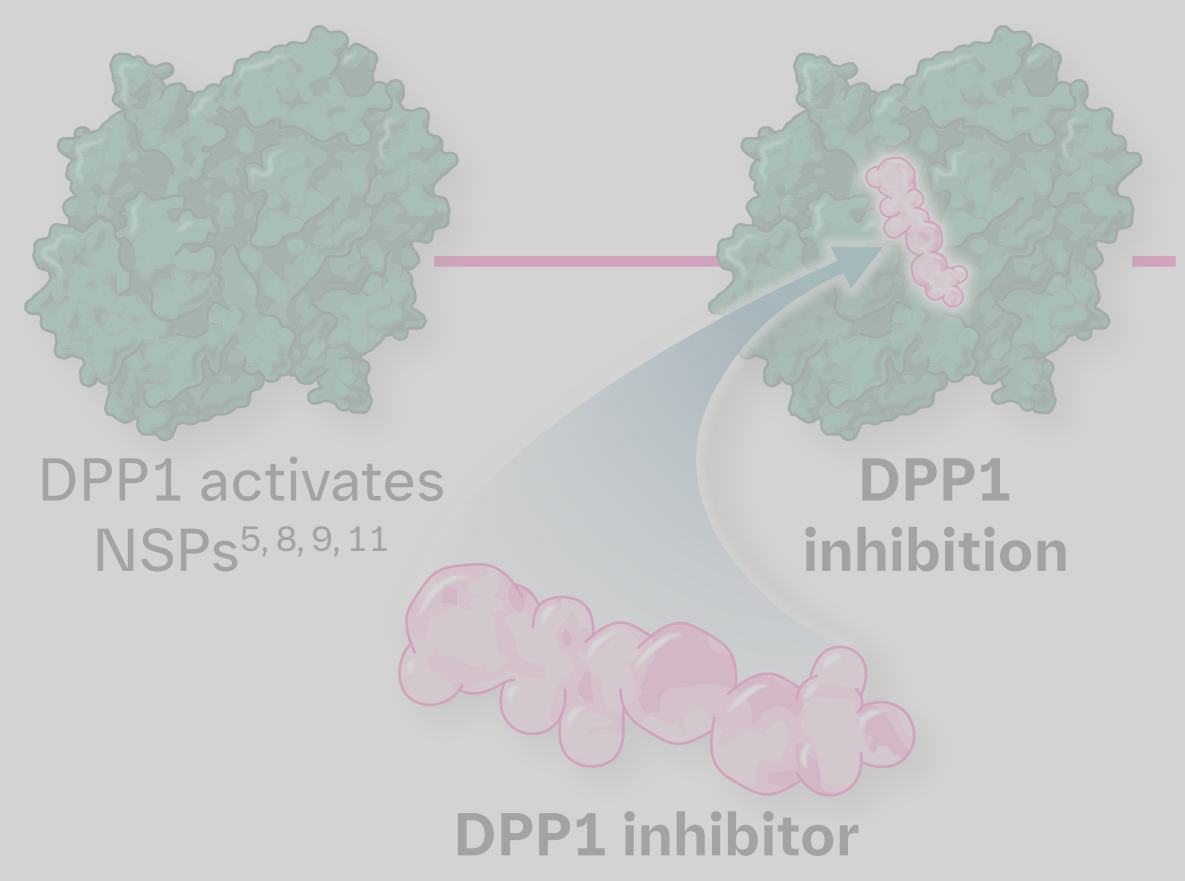


Neutrophil maturation in the bone marrow[10,11]

Fewer activated NSPs within neutrophils

Reduced imbalance between active NSPs and their inhibitors[2]

**Mitigation of downstream effects**

*Other neutrophil functions important for host defence are unimpacted*[5]

- ↓ Neutrophilic inflammation[12]
- ↑ Host defence[12]
- ↑ Mucociliary clearance[12]
- ↓ Progressive structural lung damage[12]

## DPP1 inhibitors in clinical development

**Additional efficacy and safety results**

| Compound | Phase | Study name | Additional efficacy and safety results |
|---|---|---|---|
| Brensocatib[17–19,35] (FDA & European Commission approved[13–16]) | II | WILLOW | **Efficacy:** Reductions in PEx rate (numerical for 25 mg) and dose-dependent reductions in sputum NSP activity were observed with both doses vs placebo. **Safety:** Incidence of AEs (excluding PEx) was higher with either dose than placebo. Hyperkeratosis was reported in one patient who received placebo, three patients who received the 10 mg dose of brensocatib and in one patient who received the 25 mg dose. Incidence of periodontal disease were similar across all groups |
| | IIa | NCT05090904 | **Efficacy:** Brensocatib reduced blood and sputum NSP activity **Safety:** All brensocatib doses were well tolerated, with no new identified safety signals |
| | III | ASPEN | **Efficacy:** Time to first PEx was reduced with both doses vs placebo in adults. Adults who received the 25 mg dose showed a slower decline in lung function than placebo. In adolescents, the annualised rate of PExs was numerically reduced with both doses vs placebo **Safety:** Adults had higher rates of hyperkeratosis reported in the 25 mg dose group, compared with the 10 mg dose and placebo groups. In adolescents, AEs were similar across treatment groups and consistent with overall ASPEN results |
| Florensocatib[20–22] | II | SAVE-BE | **Efficacy:** For secondary efficacy endpoints, time to first exacerbation was improved for both doses compared with placebo and there was numerical improvement in least-squares mean difference in $FEV_1$ percentage predicted compared with the placebo group for the 40 mg dose **Safety:** Safety profile of both HSK31858 doses was similar to placebo |
| | III | HOPE-BE | Yet to be published (Study completion: January 2027) |
| Verducatib[6,23–27] | II | AIRLEAF® | **Efficacy:** 2.5 mg dose reduced the risk of experiencing a severe PEx vs placebo (HR 0.12), had the largest effect on SGRQ Symptoms score and led to the greatest change in $FEV_1$ and FVC in mL. There was a numerical reduction in rate of PEx for both doses **Safety:** Verducatib demonstrated a similar safety profile to placebo, and theincidence of AEs was generally similar across groups, including potentially mode-of-action-related AEs. Hyperkeratosis was reported in 2, 0, 2, and 4 people in the placebo, 1 mg , 2.5 mg, and 5 mg dose groups, respectively |
| | IIa | CLAIRAFLY® | **Exposure/pharmacokinetics/pharmacodynamics:** A comparison between people with NCFBE from AIRLEAF® and people with CFBE from CLAIRAFLY® demonstrated verducatib exposure to be similar between the two studies for 5 mg dose of verducatib |
| | II | CLAIRLEAF® | Yet to be published (Study completion: March 2027) |
| | III | AIRTIVITY® | Yet to be published (Study completion: October 2028) |

### CFBE and CF landscape

~5–11% of people with CF are **ineligible for or intolerant of CFTR modulator therapy**[28,29]

\+

Those receiving CFTR modulator therapy have **persistent airway inflammation**[12,30–33]

=

People with CF may benefit from treatment **targeting the neutrophilic inflammation** element of their BE

**AIRTIVITY®** is the **first Phase III** DPP1 inhibitor study in BE to **include people with CF**[18,22,27]

## Evolution of DPP1 clinical studies

| | Brensocatib & florensocatib studies[17,18,22,34] | Verducatib Phase III AIRTIVITY® study[6,27] |
|---|---|---|
| | Exclusion of high-risk patients with low PEx frequency‡ | Includes people with **1 PEx** (that led to antibiotic treatment in the last 12 months before screening) and a high symptom burden§ at screening, as well as people with ≥2 PEx |
| | **Lack subgroups** such as CF, primary COPD and asthma | Includes people with **CF, COPD and asthma** |
| | **No significant effect on severe PEx** in the Phase III ASPEN study | Continuing to test the reduction in the **risk of experiencing severe PExs** seen in the Phase II AIRLEAF® study |
| | Longest treatment period in any randomised controlled DPP1 inhibitor study (excluding expanded access studies) is **52 weeks** | Treatment period of **52–76 weeks,** with potential open-label extension study |

## Conclusion

The success of brensocatib, florensocatib, and verducatib studies **validates the DPP1 inhibitor approach in BE**

Click for expert opinion piece from **Prof. James D. Chalmers**

**Does DPP1 inhibition have the potential to optimise management of BE?**
**What insights do we have on DPP1 inhibition in BE?**
**What questions remain?**

*Study is ongoing
†Including COPD and asthma
§SGRQ Symptoms score >40 for adults or according to the investigator for adolescents
‡Eligible adolescents in the brensocatib Phase III ASPEN study had at least one exacerbation that led to antibiotic treatment in the 12 months before screening[18]

**Abbreviations**

AE, adverse event; BE, bronchiectasis; CF, cystic fibrosis; CFBE, cystic fibrosis-related bronchiectasis; CFTR, cystic fibrosis transmembrane conductance regulator; COPD, chronic obstructive pulmonary disease; CT, computed tomography; DPP1, dipeptidyl peptidase 1; FDA, US Food and Drug Administration; $FEV_1$, forced expiratory volume in 1 second; FVC, forced vital capacity; HR, hazard ratio; MoA, mode of action; NCFBE, non–cystic fibrosis-related bronchiectasis; NE, neutrophil elastase; NSP, neutrophil serine protease; PEx, pulmonary exacerbation; pts, participants; SGRQ, St. George's Respiratory Questionnaire.

**References**

**1.** Long MB, et al. Lancet Respir Med. 2024; 12(11):901–14; **2.** Oriano M, et al. Int J Mol Sci. 2021; 22(11):5996; **3.** Polverino E, et al. Eur Respir J. 2017; 50(3):1700629; **4.** Flume PA, et al. Lancet. 2018; 392(10150):880–90; **5.** Chalmers JD, et al. Front Immunol. 2023; 14:1239151; **6.** Chalmers JD, et al. Eur Respir J. 2025; 65(1):2401551; **7.** Chalmers JD, et al. Eur Respir J. 2025; 65(1):2401050; **8.** Chalmers JD, et al. Am J Respir Crit Care Med. 2017; 195(10):1384–93; **9.** Adkison AM, et al. J Clin Invest. 2002; 109(3):363–71; **10.** Gardiner P, et al. Br J Pharmacol. 2016; 173(15):2390–401; **11.** Keir HR, et al. Eur Respir Rev. 2022; 31(163):210241; **12.** Mall MA, et al. Eur Respir Rev. 2024; 33(173):240001; **13.** U.S. Food & Drug Administration. BRINSUPRI™ (brensocatib) prescribing information. https://www.accessdata.fda.gov/drugsatfda_docs/label/2025/217673s000lbl.pdf. Date last accessed: 5 November 2025; **14.** European Medicines Agency. Brinsupri : EPAR - Product information. https://www.ema.europa.eu/en/documents/product-information/brinsupri-epar-product-information_en.pdf. Date last accessed: 23 January 2026; **15.** Insmed. FDA approves BRINSUPRI™ (brensocatib) as the first and only treatment for non-cystic fibrosis bronchiectasis, a serious, chronic lung disease. https://investor.insmed.com/2025-08-12-FDA-Approves-BRINSUPRI-TM-brensocatib-as-the-First-and-Only-Treatment-for-Non-Cystic-Fibrosis-Bronchiectasis,-a-Serious,-Chronic-Lung-Disease. Date last accessed: 5 November 2025; **16.** Insmed. European Commission approves BRINSUPRI™ (brensocatib) as the first and only treatment approved for non-cystic fibrosis bronchiectasis in the European Union. https://investor.insmed.com/2025-11-18-European-Commission-Approves-BRINSUPRI TM-brensocatib-as-the-first-and only treatment approved for non-cystic fibrosis bronchiectasis in the European Union. Date last accessed: 23 January 2026; **17.** Chalmers JD, et al. N Engl J Med. 2020; 383(22):2127–37; **18.** Chalmers JD, et al. N Engl J Med. 2025; 392(16):1569–81; **19.** Konstan MW, et al. Clin. Pharmacokinet. 2025 64(10):1561–74; **20.** Zhong NS, et al. Lancet Respir Med. 2025; 13(5):414–24; **21.** ClinicalTrials.gov. A study to assess the efficacy and safety of HSK31858 in participants with non-cystic fibrosis bronchiectasis. https://clinicaltrials.gov/study/NCT06660992. Date last accessed: 23 January 2026; **22.** Zhong NS, et al. ERJ Open Res. 2026; in press: https://doi.org/10.1183/23120541.00182-2026; **23.** Mall MA, et al. J Cyst Fibros. 2025; 24:S35; **24.** ClinicalTrials.gov. A study to test how well BI 1291583 is tolerated by people with cystic fibrosis bronchiectasis (Clairafly™). https://www.clinicaltrials.gov/study/NCT05865886. Date last accessed: 26 January 2026; **25.** ClinicalTrials.gov. Clairleaf™: A study to test long-term treatment with BI 1291583 in people with bronchiectasis who took part in a previous study with this medicine. https://clinicaltrials.gov/study/NCT05846230. Date last accessed: 23 January 2026; **26.** ClinicalTrials.gov. The AIRTIVITY™ study: A study to find out whether BI 1291583 helps people with bronchiectasis. https://www.clinicaltrials.gov/study/NCT06872892. Date last accessed: 26 January 2026; **27.** Chalmers JD, et al. ERJ Open Res. 2026; in press: https://doi.org/10.1183/23120541.01068-2025; **28.** Burgel P-R, Mall MA. Eur Respir J 2026; 67: 2501783; **29.** Cystic Fibrosis Foundation. 2024 Patient Registry Annual Data Report. Bethesda, Maryland: Cystic Fibrosis Foundation; 2025; **30.** Schaupp L, et al. Eur Respir J. 2023; 62(2):2202153; **31.** Casey M, et al. Thorax. 2023; 78(8):835–9; **32.** Nussstein H, et al. Eur Respir J. 2025; 2026; 67(2):2500616; **33.** Sagel SD, et al. Ann Am Thorac Soc. 2025; online ahead of print: https://doi.org/10.1513/AnnalsATS.202507-817OC; **34.** Chalmers JD, et al. ERJ Open Res. 2024; 10(4):00151-2024; **35.** Maitre B, et al. Am J Respir Crit Care Med. 2025; 211:A7421; **36.** Cipolla D, et al. Respir Res. 2023; 24(1):133.

**Acknowledgements**

This interactive PDF was funded by Boehringer Ingelheim Pharmaceutical Inc. The authors meet criteria for authorship as recommended by the International Committee of Medical Journal Editors (ICMJE). The authors did not receive payment related to the development of the interactive PDF. Amy Bohm, MBio, of Nucleus Global, provided writing, editorial support and formatting assistance, which was contracted and funded by Boehringer Ingelheim. Boehringer Ingelheim was given the opportunity to review the interactive PDF for medical and scientific accuracy as well as intellectual property considerations.

# The dipeptidyl peptidase 1 inhibition landscape in bronchiectasis and its future direction

Chalmers JD, Jain R, O'Donnell AE, *et al*. The dipeptidyl peptidase 1 inhibition landscape in bronchiectasis and its future direction. *Eur Respir Rev* 2026; 35: 260213. doi: 10.1183/16000617.0213-2026.

## Introduction to bronchiectasis and dipeptidyl peptidase 1 inhibition

**BE and DPP1 inhibition**

BE is a chronic lung disease characterised by **neutrophilic inflammation**[1–4]

Pharmacological **inhibition of DPP1** aims to suppress NSP activity[5–9]

To reduce PEx frequency and severity, and improve lung function in people with BE[7,10]

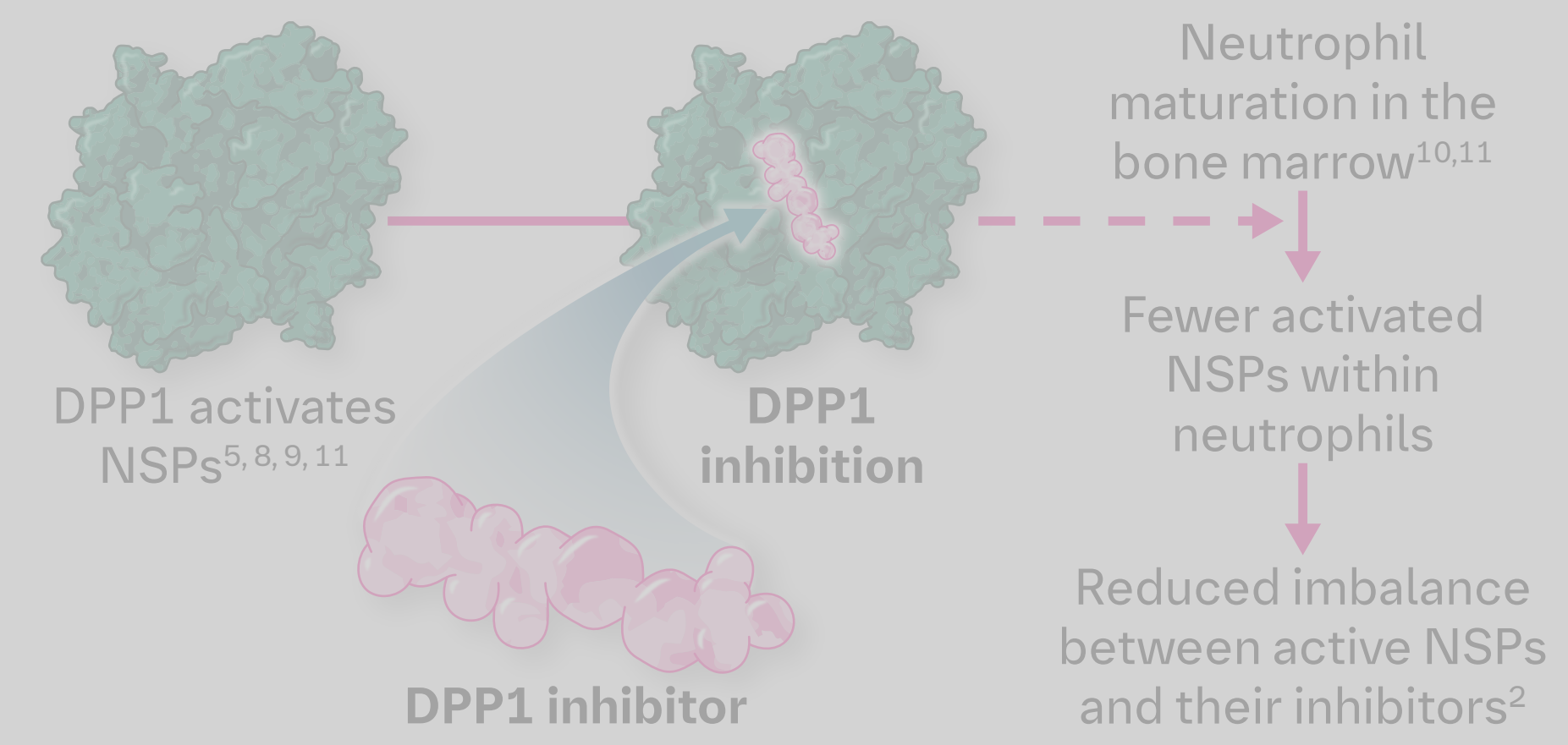


**Mitigation of downstream effects**

*Other neutrophil functions important for host defence are unimpacted*[6]

- Neutrophilic inflammation[12] (decreased)
- Host defence[12] (increased)
- Mucociliary clearance[12] (increased)
- Progressive structural lung damage[12] (decreased)

### Expert opinion: Prof. James D. Chalmers

DPP1 inhibition represents a paradigm shift in the management of patients with BE. Treatments to date have predominantly focused on palliating symptoms (airway clearance, mucoactive drugs) or targeting infection with antibiotics. Even macrolides, the most effective current treatment, is an antibiotic class and runs the risk of driving antimicrobial resistance, and has a poorly defined mechanism of action. As we think about an optimal treatment for BE, we would wish to have a treatment which can reduce the burden of exacerbations and symptoms, which addresses the inflammatory process that is central to the disease, and which can interrupt the "vicious vortex" of disease without carrying the long-term risks associated with repeated broad spectrum antibiotic exposure. DPP1 inhibition achieves these objectives. DPP1 inhibition therefore has the potential to optimise the management of patients with BE at high risk of exacerbations. The evidence from Phase II and Phase III trials on exacerbations is compelling with approximate efficacy of 20–60% reduction on key exacerbation endpoints across the different compounds. That all trials to date have met their primary endpoints strongly validates the mechanism.

There are several compelling findings with DPP1 inhibitors worthy of discussion. First, DPP1 inhibition has a remarkable effect on the "vicious vortex" of BE. Translational research has shown a strong effect of DPP1 inhibition on reducing MUC5AC levels in sputum. Reductions in this key inflammatory mucin may underlie the clinical efficacy of DPP1 inhibition in BE, since mucus symptoms are dominant in the disease and are central to lung function decline. This effect on mucociliary clearance is supported by recently reported data from the CT substudy of the ASPEN trial suggesting reduced mucus plugging. DPP1 inhibition enhances antimicrobial defence by upregulating the secretory leukocyte protease inhibitor and neutrophil defensins as well as resetting the cytokine response. These effects likely underlie the beneficial effects on lung function decline seen with the 25 mg dose of brensocatib, and it was observed that 25 mg had a greater impact on MUC5AC than 10 mg.

The ability to prevent progression of disease as measured by $FEV_1$, FVC, and in an exploratory analysis, to reduce mucus plugging on CT, raises the potential of DPP1 inhibition as a disease-modifying therapy. Disease modification is defined as delaying, slowing, or preventing the progression of a disease by targeting its underlying cause rather than merely alleviating symptoms. Progression of bronchiectasis is not well defined but most definitions would include changes over time in lung function or radiological appearance on CT, both of which are altered by DPP1 inhibitors. Longer-term studies beyond 1 year are likely needed to determine whether the apparent slowing of disease progression with these therapies is sustained.

Another remarkable observation is the consistency of effect across patient subgroups. In WILLOW, ASPEN, AIRLEAF®, and SAVE-BE, no "non-responding subgroup" has been consistently identified, and perhaps only patients from Asian countries have been proposed as super responders. This appears to confirm the central role of neutrophilic inflammation in BE. The target is present in all patients and therefore the benefit is possible in all patient groups.

The potential disease-modifying nature of the treatment is, in my experience, important for patients who want a treatment that will make them feel better, prevent exacerbations, but also want a treatment that will prevent the disease from getting worse.

Nevertheless, this raises a number of difficult questions to answer. Where should DPP1 inhibition be positioned in relation to other therapies such as macrolides? What does success look like and at what stage should treatment success be evaluated? Upcoming guidelines that includes DPP1 inhibition may address some of these questions, which are likely to be different in different healthcare systems. An important shift in thinking is needed towards an idea of disease activity - where the goal of treatment is to achieve disease control/stability, represented by a low risk of exacerbation, stable lung function, and manageable symptoms.

My personal view is that for patients at high risk of exacerbations and progressive disease, DPP1 inhibition represents the most effective evidence-based therapy and has the potential to become the backbone therapy for "highly active disease". The DPP1 inhibition landscape is evolving, as is our understanding of the disease. Next-generation DPP1 inhibitors will broaden our understanding of the role of these drugs by broadening the patient population in which they have been tested, the length of time over which they are tested, and the endpoints tested. This may allow more patients to have access to these therapies as well as strengthening the evidence of whether DPP1 inhibitors are genuinely disease modifying. Any differentiation between the drugs is speculative and will need to be established in Phase III or real-life studies.

Taken together, DPP1 inhibition represents an exciting development for BE patients who continue to have a massive unmet need. These are new therapies and advancing the science in this area is critical if this new tool is to be used optimally.

| | |
|---|---|
| Exclusion of high-risk patients with low PEx frequency‡ | treatment in the last 12 months before screening) and a high symptom burden§ at screening, as well as people with ≥2 PEx |
| **Lack subgroups** such as CF, primary COPD and asthma | Includes people with **CF, COPD and asthma** |
| **No significant effect on severe PEx** in the Phase III ASPEN study | Continuing to test the reduction in the **risk of experiencing severe PExs** seen in the Phase II AIRLEAF® study |
| Longest treatment period in any randomised controlled DPP1 inhibitor study (excluding expanded access studies) is **52 weeks** | Treatment period of **52–76 weeks,** with potential open-label extension study |

## Conclusion

The success of brensocatib, florensocatib, and verducatib studies **validates the DPP1 inhibitor approach in BE**

Click for expert opinion piece from **Prof. James D. Chalmers**

**Does DPP1 inhibition have the potential to optimise management of BE?**
**What insights do we have on DPP1 inhibition in BE?**
**What questions remain?**

*Study is ongoing
†Including COPD and asthma
§SGRQ Symptoms score >40 for adults or according to the investigator for adolescents
‡Eligible adolescents in the brensocatib Phase III ASPEN study had at least one exacerbation that led to antibiotic treatment in the 12 months before screening[18]

**Abbreviations**

AE, adverse event; BE, bronchiectasis; CF, cystic fibrosis; CFBE, cystic fibrosis-related bronchiectasis; CFTR, cystic fibrosis transmembrane conductance regulator; COPD, chronic obstructive pulmonary disease; CT, computed tomography; DPP1, dipeptidyl peptidase 1; FDA, US Food and Drug Administration; $FEV_1$, forced expiratory volume in 1 second; FVC, forced vital capacity; HR, hazard ratio; MoA, mode of action; NCFBE, non–cystic fibrosis-related bronchiectasis; NE, neutrophil elastase; NSP, neutrophil serine protease; PEx, pulmonary exacerbation; pts, participants; SGRQ, St. George's Respiratory Questionnaire.

**References**

**1.** Long MB, et al. Lancet Respir Med. 2024; 12(11):901–14; **2.** Oriano M, et al. Int J Mol Sci. 2021; 22(11):5996; **3.** Polverino E, et al. Eur Respir J. 2017; 50(3):1700629; **4.** Flume PA, et al. Lancet. 2018; 392(10150):880–90; **5.** Chalmers JD, et al. Front Immunol. 2023; 14:1239151; **6.** Chalmers JD, et al. Eur Respir J. 2025; 65(1):2401551; **7.** Chalmers JD, et al. Eur Respir J. 2025; 65(1):2401050; **8.** Chalmers JD, et al. Am J Respir Crit Care Med. 2017; 195(10):1384–93; **9.** Adkison AM, et al. J Clin Invest. 2002; 109(3):363–71; **10.** Gardiner P, et al. Br J Pharmacol. 2016; 173(15):2390–401; **11.** Keir HR, et al. Eur Respir Rev. 2022; 31(163):210241; **12.** Mall MA, et al. Eur Respir Rev. 2024; 33(173):240001; **13.** U.S. Food & Drug Administration. BRINSUPRI™ (brensocatib) prescribing information. https://www.accessdata.fda.gov/drugsatfda_docs/label/2025/217673s000lbl.pdf. Date last accessed: 5 November 2025; **14.** European Medicines Agency. Brinsupri : EPAR - Product information. https://www.ema.europa.eu/en/documents/product-information/brinsupri-epar-product-information_en.pdf. Date last accessed: 23 January 2026; **15.** Insmed. FDA approves BRINSUPRI™ (brensocatib) as the first and only treatment for non-cystic fibrosis bronchiectasis, a serious, chronic lung disease. https://investor.insmed.com/2025-08-12-FDA-Approves-BRINSUPRI-TM-brensocatib-as-the-First-and-Only-Treatment-for-Non-Cystic-Fibrosis-Bronchiectasis,-a-Serious,-Chronic-Lung-Disease. Date last accessed: 5 November 2025; **16.** Insmed. European Commission approves BRINSUPRI™ (brensocatib) as the first and only treatment approved for non-cystic fibrosis bronchiectasis in the European Union. https://investor.insmed.com/2025-11-18-European-Commission-Approves-BRINSUPRI TM-brensocatib-as-the-first-and only treatment approved for non-cystic fibrosis bronchiectasis in the European Union. Date last accessed: 23 January 2026; **17.** Chalmers JD, et al. N Engl J Med. 2020; 383(22):2127–37; **18.** Chalmers JD, et al. N Engl J Med. 2025; 392(16):1569–81; **19.** Konstan MW, et al. Clin. Pharmacokinet. 2025 64(10):1561–74; **20.** Zhong NS, et al. Lancet Respir Med. 2025; 13(5):414–24; **21.** ClinicalTrials.gov. A study to assess the efficacy and safety of HSK31858 in participants with non-cystic fibrosis bronchiectasis. https://clinicaltrials.gov/study/NCT06660992. Date last accessed: 23 January 2026; **22.** Zhong NS, et al. ERJ Open Res. 2026; in press: https://doi.org/10.1183/23120541.00182-2026; **23.** Mall MA, et al. J Cyst Fibros. 2025; 24:S35; **24.** ClinicalTrials.gov. A study to test how well BI 1291583 is tolerated by people with cystic fibrosis bronchiectasis (Clairafly™). https://www.clinicaltrials.gov/study/NCT05865886. Date last accessed: 26 January 2026; **25.** ClinicalTrials.gov. Clairleaf™: A study to test long-term treatment with BI 1291583 in people with bronchiectasis who took part in a previous study with this medicine. https://clinicaltrials.gov/study/NCT05846230. Date last accessed: 23 January 2026; **26.** ClinicalTrials.gov. The AIRTIVITY™ study: A study to find out whether BI 1291583 helps people with bronchiectasis. https://www.clinicaltrials.gov/study/NCT06872892. Date last accessed: 26 January 2026; **27.** Chalmers JD, et al. ERJ Open Res. 2026; in press: https://doi.org/10.1183/23120541.01068-2025; **28.** Burgel P-R, Mall MA. Eur Respir J 2026; 67: 2501783; **29.** Cystic Fibrosis Foundation. 2024 Patient Registry Annual Data Report. Bethesda, Maryland: Cystic Fibrosis Foundation; 2025; **30.** Schaupp L, et al. Eur Respir J. 2023; 62(2):2202153; **31.** Casey M, et al. Thorax. 2023; 78(8):835–9; **32.** Nussstein H, et al. Eur Respir J. 2025; 2026; 67(2):2500616; **33.** Sagel SD, et al. Ann Am Thorac Soc. 2025; online ahead of print: https://doi.org/10.1513/AnnalsATS.202507-817OC; **34.** Chalmers JD, et al. ERJ Open Res. 2024; 10(4):00151-2024; **35.** Maitre B, et al. Am J Respir Crit Care Med. 2025; 211:A7421; **36.** Cipolla D, et al. Respir Res. 2023; 24(1):133.

**Acknowledgements**

This interactive PDF was funded by Boehringer Ingelheim Pharmaceutical Inc. The authors meet criteria for authorship as recommended by the International Committee of Medical Journal Editors (ICMJE). The authors did not receive payment related to the development of the interactive PDF. Amy Bohm, MBio, of Nucleus Global, provided writing, editorial support and formatting assistance, which was contracted and funded by Boehringer Ingelheim. Boehringer Ingelheim was given the opportunity to review the interactive PDF for medical and scientific accuracy as well as intellectual property considerations.
